# Mini review: Current status and perspective of S100B protein as a biomarker in daily clinical practice for diagnosis and prognosticating of clinical outcome in patients with neurological diseases with focus on acute brain injury

**DOI:** 10.1186/s12868-023-00807-2

**Published:** 2023-07-20

**Authors:** Tammam Abboud, Veit Rohde, Dorothee Mielke

**Affiliations:** Department of Neurosurgery, University Medical Center Göttingen, Robert-Koch-Straße 40, 37075 Göttingen, Germany

**Keywords:** S100B, Traumatic brain injury, Subarachnoid hemorrhage

## Abstract

Prognosticating the clinical outcome of neurological diseases is essential to guide treatment and facilitate decision-making. It usually depends on clinical and radiological findings. Biomarkers have been suggested to support this process, as they are deemed objective measures and can express the extent of tissue damage or reflect the degree of inflammation. Some of them are specific, and some are not. Few of them, however, reached the stage of daily application in clinical practice. This mini review covers available applications of the S100B protein in prognosticating clinical outcome in patients with various neurological disorders, particularly in those with traumatic brain injury, spontaneous subarachnoid hemorrhage and ischemic stroke. The aim is to provide an understandable picture of the clinical use of the S100B protein and give a brief overview of the current limitations that require future solutions.

## Introduction

Modern medical practice increasingly depends on laboratory and instrumental diagnostic procedures. This can be attributed to rapid technological advances and often huge workloads [1] that limit the time needed for clinical assessment in busy units. In addition, noninvestigator- dependent diagnostic tools can be more objective in certain aspects [2] and could facilitate the emerging application of telemedicine [3]. In many internal diseases, physicians use biomarkers for diagnostics and monitoring of the clinical course of the disease. In contrast, the application of biomarkers in neurological diagnosis has been less familiar, probably because the blood brain barrier was thought to restrict the ability of biomarkers to enter peripheral blood and be measured. However, modern techniques have enabled the detection of such biomarkers and thus allowed for their application mostly in clinical studies. Some of these biomarkers have been under investigation for decades [4, 5], and others are emerging [6]. In addition, biomarkers such as tau and blood neurofilament light protein were shown to be useful in the specific field of neurodegenerative diseases [7], while others, including S100B, covers a wider spectrum of neurological diseases, including traumatic brain injury, subarachnoid hemorrhage, epilepsy and neurodegenerative diseases [8, 9]. This mini review will address the most common applications of S100B in prognosticating clinical outcome in patients with neurological diseases in general and underline its role in acute diagnostics in patients with traumatic brain injury, spontaneous subarachnoid hemorrhage and stroke. It will also refer to current limitations and future solutions in clinical practice. In general, this review will deal with S100B in serum and a separate section will address S100B in cerebrospinal fluid (CSF). Although S100B can be found in other biological fluids such as amniotic fluid, urine and saliva [10], which might be of use in perinatal and pediatric disorders, these aspects will not be discussed in this review.

## S100B as an inflammatory measure of the brain

S100B is a calcium-binding protein, widely expressed in the brain predominantly by astrocytes, but also by maturing oligodendrocytes, neural progenitor cells, dendritic cells and specific lymphocyte subpopulations. S100B can be released extracellularly and is considered as a marker of brain damage [11]. Many studies have related S100B to a diversity of psychiatric and neurodegenerative diseases, such as Alzheimer’s disease, Down’s syndrome, schizophrenia, and Tourette’s syndrome, in addition to the known correlation between S100B and trauma and stroke. These broad implications have led to the proposal that S100B is the CRP (C-reactive protein) of the brain [5].

The potential use of S100B as a biomarker ranges from determining the severity of a certain disease and predicting the clinical outcome to monitoring disease progression and response to treatment. The type of application differs according to the disease features being chronic such as neurodegenerative diseases and other neurological and psychiatric disorders, or acute such as subarachnoid hemorrhage, trauma, and stroke. It is noteworthy that S100B has been suggested to be involved in the pathogenesis of some neurological diseases, such as Parkinson’s disease [11] and multiple sclerosis [12], making it a possible treatment target. However, therapeutic approaches still have a long way to go. Michetti et al. reported that there is mounting evidence pointing to S100B as a Damage-Associated Molecular Pattern molecule [13] and that in many cases its over-expression leads to disease worsening [10]. They also argued that in experimental models of diseases such as Alzheimer’s and Parkinson’s diseases, amyotrophic lateral sclerosis, multiple sclerosis, traumatic and vascular acute neural injury, epilepsy, and inflammatory bowel disease, changes of S100B levels are associated with the occurrence of clinical and/or toxic parameters [13]. Yet, drugs that counteract S100B are still experimental, and clinical studies are needed to validate this aspect.

## S100B for diagnostics and treatment evaluation in chronic neurological and psychiatric diseases

There is evidence for a significant increase in serum S100B levels following the onset of symptoms in patients with migraine and epilepsy [14–16]. However, a clinical application in terms of predicting the onset of seizures or migraine attacks or evaluating the response to medication has not yet been demonstrated.

In cases of suspected neurodegenerative disorders, reliance on S100B concentrations for differential diagnostic purposes is not recommended due to lack of disease specificity. Moreover, there is no consistent evidence for a correlation between disease severity and concentrations of S100B in serum. Therefore, S100B has limited usefulness for monitoring disease progression [17]. In addition, imaging biomarkers such as PET imaging of Alzheimer’s disease tau pathology are currently gaining more attention in the diagnosis and monitoring of neurodegenerative diseases along with blood-based biomarkers such as amyloid-β, phosphorylated tau and neurofilament light protein [7, 18].

Many studies have provided evidence that peripheral S100B concentrations are elevated in individuals diagnosed with schizophrenia when compared with healthy controls 19; however, no difference could be found between drug-free patients and patients on antipsychotic medication [20]. Similarly, Dung et al. found higher plasma concentrations of S100B in patients with schizophrenia than in healthy controls, while no significant correlations between S100B concentrations and psychotic symptoms or cognition could be detected within the patient group [21]. In affective disorders, including major depressive episodes in patients with major depressive disorder and both manic and depressive episodes in patients with bipolar disorder, elevated S100B levels might be associated with mood episodes and were consistently elevated in patients with acute affective episodes in comparison to healthy controls [22]. It has been demonstrated that S100B is more strongly elevated in major depressive disorder than in bipolar disorder. Successful antidepressive treatment has been associated with serum S100B reduction in major depression, whereas there is no evidence of treatment effects in mania [23]. Interestingly, lower S100B serum levels also seem to be of some diagnostic value, as was found in a recent study that investigated the combination of S100B and cytokines in determining generalized anxiety disorder and demonstrated a diagnostic accuracy of 94% [24].

## Traumatic brain injury

S100B is the only single biomarker with a validated clinically useful cutoff value in mild traumatic brain injury (TBI) [25]. The Scandinavian Neurotrauma Committee has recommended the use of serum S100B as a biomarker for mild low-risk TBI [26]. Specifically, its high sensitivity allows serum S100B under the cutoff value (0.10 µg/L), when determined within 6 h following trauma, to rule out serious traumatic intracranial hemorrhage in adult patients with mild head injury and without other risk factors [27]. Based on that, physicians can omit performing CT scans in those patients, which in turn reduces treatment costs and alleviates the logistic burden. In this regard, it is noteworthy to mention that Biberthaler et al. found that adding the measurement of S100B concentration to the clinical decision rules for a CT scan in patients with minor head injury could allow a 30% reduction in scans [28]. It would be desirable to use S100B to reduce CT scans in children with mild TBI. Although S100 B serum concentrations increases in children following trauma [29], S100B doesn’t seem to discriminate between symptomatic and asymptomatic children with minor head injury [30] and it is in general not a reliable prognostic index in paediatric TBI [31]. In addition, some reports suggested that serum S100B might be useful in screening for sport-related concussions [32, 33], others, however, could not confirm that [34].

The relatively low specificity of S100B limits its role in patients with moderate or severe brain trauma or even with multiple traumas. On the one hand, Müller et al. reported that fractures and thoracic injuries appeared to be the main factors associated with increased S100B levels and that head injury may only play a minor role in S100B serum elevation in multiple trauma patients [35]. On the other hand, they suggested that normal S100 B has a good negative predictive value for in-hospital mortality and that S100B levels were associated with trauma severity and might thus be of use as a prognostic marker in trauma patients [35]. Indeed, higher serum values of S100B in the first days after severe TBI seem to correlate with mortality [36]. However, to date, there is no established cutoff threshold to identify patients without survival potential. Such a cutoff value with sufficient specificity is a precondition for a biomarker to be able to support the decision-making process.

## Spontaneous subarachnoid hemorrhage

Spontaneous subarachnoid hemorrhage (SAH) is usually associated with damage to the central nervous system. Although other organs can be involved in the acute onset of the disease, primary cell damage is expected to take place at the level of neural tissue. Based on that, an elevation in biomarker levels is usually specific to neural damage. This was the case for many biomarkers that were investigated in the context of SAH including H-FABP, S100B, troponin I, NDKA, UFD-1, neuron-specific enolase and neurofilament light chain [9, 37–39]. Higher values of S100B were found to correlate with mortality and unfavorable outcome 9, 37, 40, 41; and the prognostic value of S100B seemed to be superior to that of other markers [42].

Patients with severe SAH, such as those with severe TBI, face a significant short- and long-term mortality risk and pose a similar diagnostic and decision-making dilemma. However, secondary complications, mainly vasospasm and delayed cerebral ischemia, occur later in the course of SAH, which might reduce the sensitivity of biomarkers for prognostication of long-term clinical outcome when measured within the first days after hemorrhage but would not affect their specificity regarding prediction of poor outcome [9]. Aineskog et al. found a correlation between S100B serum levels and poor clinical outcome one year after SAH with 95% specificity [43]. The question remains, however, whether clinical decision-making can rely on such criteria in the early stage of the disease.

## Ischemic stroke

Serum S100B has been extensively investigated in ischemic stroke patients. This includes acute diagnostic, infarct volume, development of intracranial hemorrhage, monitoring of treatment success and outcome prediction. Regarding acute diagnostic, S100B has a low specificity for ischemic stroke and its serum levels doe not seem to increase immediately after acute stroke and might reach the highest values at day 3 after symptom onset [44]. S100B serum levels, however, seem to be able to predict stroke complications. In particular, they correlate well with infarct volume [44, 45] and can predict a malignant course of infarction in proximal occlusion of medial cerebral artery [46]. Moreover, serum S100B levels measured within 24 h after symptom onset were independently associated with the development of symptomatic intracranial hemorrhage and symptomatic brain edema in acute ischemic stroke patients [47]. Despite that, S100B is not deemed a reliable biomarker to predict hemorrhagic transformation in thrombolyzed patients with stroke in clinical practice, mainly because its low diagnostic accuracy [48]. Interestingly, S100B is a promising biomarker for control of treatment success after clot lysis or mechanical thrombectomy. In a relatively small cohort of 23 patients, Foerch et al. found that a single S100B value < 0.4 µg/L obtained 48–96 h after stroke onset indicates successful clot lysis < 6 h in proximal occlusion of medial cerebral artery with a high degree of accuracy [49]. Unfortunately, we couldn’t find a further validation of this cutoff value in larger series or multicentric studies. Luger et al. reported a class I evidence that S100B 2 days following mechanical thrombectomy for acute ischemic stroke accurately distinguishes favorable from unfavorable functional outcome [50]. Without determining a cutoff value, they found that successful recanalization resulted both in low S100B values in patients in whom the intervention prevented the development of a final infarct, and in high S100B values in patients who developed infarcts despite recanalization.

Similar to traumatic brain injury and subarachnoid hemorrhage, functional recovery of patients with ischemic stroke can be predicted by measuring serum S100B. Its concentrations at days 2 to 4 after acute stroke may predict neurological status as well as functional impairment at discharge [51].

## S100B in cerebrospinal fluid

Because S100B elevation in serum can have extra-cranial sources [35], S100B in CSF was thought to be a reasonable alternative. Böhmer et al. studied CSF S100B in twenty patients with severe TBI and found that up to 3 days, CSF S100B levels were significantly elevated in patients who died following trauma compared to those who survived [52]. Kellermann et al. compared between serum and CSF concentrations of S100B in TBI and SAH patients. They found that peak S100B levels in CSF and serum were measured on the first day after head trauma and concentrations decreased during the ensuing days post injury gradually. Both in TBI and SAH patients S100B concentrations in CSF and serum were significantly higher in patients with an unfavorable outcome in comparison to patients with a good outcome [53]. However, they suggested that initial S100B levels in CSF have a limited prognostic value in neurotrauma, since they are highly sensitive to smallest external influences like incretion of ventricle drainage [53]. We add to this concern that, unless there is already a CSF drainage, obtaining CSF in patients with severe SAH or TBI and consequently pathological intracranial pressure, might be problematic and poses a further limitation to the role of CSF S100B in these diseases.

Similar to serum S100B, CSF S100B is elevated in frontotemporal lobe dementia [54] and earlier stages of Alzheimer’s disease [55, 56]. It is also significantly increased in patients with Parkinson’s disease comparing to healthy controls and correlates positively with disease severity [57]. Thus, because it can be elevated in several diseases, CSF S100B is considered a non-specific marker in neurodegenerative disorders [57]. We couldn’t find reports on the role of CSF S100B in treatment evaluation in patients with neurodegenerative diseases.

## Limitations and future solutions

Many aspects limit the usability of S100B as a surrogate marker. One is that S100B is not brain-specific because of the wide extra-astrocytic and extracerebral expression of S100B [17]. In addition, most of the published studies thus far have been single-centered [17]. The major reason why S100B has not yet entered medical daily practice is probably that apart from guidelines for the treatment of traumatic brain injury, which has been discussed before, none of the existing treatment guidelines involve or even suggest the use of S100B as a diagnostic adjunct. This fact applies for all neurological disorders other than trauma, in which S100B might play a diagnostic role, including subarachnoid hemorrhage [58], Ischemic stroke [59], migraine [60], schizophrenia [61], Alzheimer [62] and epilepsy (see Evidence-Based Guidelines for epilepsy as proposed by the American Epilepsy Society, https://www.aesnet.org/clinical-care/clinical-guidance/guidelines). For S100B to be adopted by clinical guidelines as a surrogate marker, a higher level of evidence will be needed. Large-scale studies and national registries might provide the basis for such evidence. Moreover, because there are other markers that compete with S100B, the use of artificial intelligence might be helpful to build a marker hierarchy or perhaps to combine different markers to reach a higher sensitivity and specificity in prognosticating clinical outcome.

## Data Availability

Not applicable.
